# Alkaline Liquid Ventilation of the Membrane Lung for Extracorporeal Carbon Dioxide Removal (ECCO_2_R): In Vitro Study

**DOI:** 10.3390/membranes11070464

**Published:** 2021-06-22

**Authors:** Luigi Vivona, Michele Battistin, Eleonora Carlesso, Thomas Langer, Carlo Valsecchi, Sebastiano Maria Colombo, Serena Todaro, Stefano Gatti, Gaetano Florio, Antonio Pesenti, Giacomo Grasselli, Alberto Zanella

**Affiliations:** 1 Anesthesia and Critical Care, Department of Pathophysiology and Transplantation, University of Milan, 20122 Milan, Italy; luigi.vivona@unimi.it (L.V.); eleonora.carlesso@unimi.it (E.C.); sebastiano.colombo@gmail.com (S.M.C.); serena.todaro@outlook.it (S.T.); gaetano.florio@unimi.it (G.F.); antonio.pesenti@unimi.it (A.P.); giacomo.grasselli@unimi.it (G.G.); alberto.zanella1@unimi.it (A.Z.); 2 Center for Preclinical Research, Fondazione IRCCS Ca’ Granda-Ospedale Maggiore Policlinico, 20122 Milan, Italy; battistin.michele@gmail.com (M.B.); stefano.gatti@policlinico.mi.it (S.G.); 3 Department of Anesthesia and Intensive Care Medicine, Niguarda Ca’ Granda, 20162 Milan, Italy; 4 Department of Medicine and Surgery, University of Milan-Bicocca, 20900 Monza, Italy; 5 Dipartimento di Anestesia, Rianimazione ed Emergenza Urgenza, Fondazione IRCCS Ca’ Granda-Ospedale Maggiore Policlinico, 20122 Milan, Italy; carlovalsecchi5@gmail.com

**Keywords:** extracorporeal CO_2_ removal, liquid ventilation, membrane lung

## Abstract

Extracorporeal carbon dioxide removal (ECCO_2_R) is a promising strategy to manage acute respiratory failure. We hypothesized that ECCO_2_R could be enhanced by ventilating the membrane lung with a sodium hydroxide (NaOH) solution with high CO_2_ absorbing capacity. A computed mathematical model was implemented to assess NaOH–CO_2_ interactions. Subsequently, we compared NaOH infusion, named “alkaline liquid ventilation”, to conventional oxygen sweeping flows. We built an extracorporeal circuit with two polypropylene membrane lungs, one to remove CO_2_ and the other to maintain a constant PCO_2_ (60 ± 2 mmHg). The circuit was primed with swine blood. Blood flow was 500 mL × min^−1^. After testing the safety and feasibility of increasing concentrations of aqueous NaOH (up to 100 mmol × L^−1^), the CO_2_ removal capacity of sweeping oxygen was compared to that of 100 mmol × L^−1^ NaOH. We performed six experiments to randomly test four sweep flows (100, 250, 500, 1000 mL × min^−1^) for each fluid plus 10 L × min^−1^ oxygen. Alkaline liquid ventilation proved to be feasible and safe. No damages or hemolysis were detected. NaOH showed higher CO_2_ removal capacity compared to oxygen for flows up to 1 L × min^−1^. However, the highest CO_2_ extraction power exerted by NaOH was comparable to that of 10 L × min^−1^ oxygen. Further studies with dedicated devices are required to exploit potential clinical applications of alkaline liquid ventilation.

## 1. Introduction

Extracorporeal carbon dioxide removal (ECCO_2_R) clears CO_2_ from the blood through an extracorporeal membrane lung (ML). This allows independent modulation of minute ventilation and arterial partial pressure of CO_2_ (PaCO_2_), which are otherwise physiologically linked [1]. ECCO_2_R has been proposed to facilitate ultra-protective ventilation [2,3,4] and to promote non-invasive ventilation [5]. This could be particularly beneficial in patients suffering from respiratory failure, including exacerbations of chronic obstructive pulmonary disease (COPD) [6], acute respiratory distress syndrome (ARDS) [7], and patients awaiting lung transplantation [8]. The amount of CO_2_ removed by the extracorporeal support is a crucial determinant of clinical efficacy [9,10]. However, the clinical benefits of ECCO_2_R are still under evaluation due to safety concerns, mainly related to hemorrhagic and thrombotic adverse events [9].

Several ECCO_2_R devices are clinically available. They are mainly characterized by a low extracorporeal blood flow (i.e., <500 mL × min^−1^) to achieve minimally invasive approaches [11]. Indeed, although 500 mL of blood contain an amount of CO_2_ comparable to the amount of CO_2_ produced by the body in one minute ($\dot{V}$CO_2_), the relatively low CO_2_ transfer efficiency of conventional MLs significantly reduces the efficacy of these strategies [12].

The transmembrane gradient of PCO_2_ is the driving force that moves CO_2_ from blood to the sweeping gases. However, the use of high sweep gas flows, while maximizing the transmembrane gradient, does not increase CO_2_ clearance significantly. Indeed, during ECCO_2_R, most of the extracorporeal CO_2_ removal capacity is achieved for sweep gas flows below 2 L × min^−1^ since, at higher flows, the system rapidly loses efficiency [13,14,15,16].

Several ECCO_2_R techniques are currently undergoing preclinical evaluations. The main aim is to overcome the present limitations to enhance CO_2_ removal [17,18,19,20,21] effectively. To this purpose, our group has achieved high rates of CO_2_ removal through acidification of the blood entering the ML [22,23,24,25,26,27]. This strategy reduced dissociated CO_2_ (HCO_3_^−^) in favor of dissolved CO_2_ (PCO_2_), thus increasing the efficiency of ECCO_2_R. Nevertheless, these approaches are still experimental, mainly due to safety and technical issues [28,29].

In the present study, we hypothesized that extracorporeal CO_2_ removal could be enhanced through the ventilation of the ML with a sweep fluid with an extremely high CO_2_ absorbing capacity (sodium hydroxide -NaOH- solutions), thereby preserving the transmembrane CO_2_ gradient.

Indeed, when a high amount of CO_2_ is added to dilute NaOH solutions, carbon dioxide first hydrates to carbonic acid (H_2_CO_3_), Equation (1), which will subsequently react with NaOH to form sodium bicarbonate (NaHCO_3_), Equation (2).

CO_2_ + H_2_O ↔ H_2_CO_3_ (1)

NaOH + H_2_CO_3_ ↔ Na^+^ + HCO_3_^−^ + H_2_O (2)

Instead, when CO_2_ is added to highly concentrated NaOH solutions, sodium bicarbonate is formed directly, Equation (3), which subsequently forms sodium carbonate, Equation (4).

NaOH + CO_2_ ↔ NaHCO_3_ (3)

NaHCO_3_ + NaOH ↔ Na_2_CO_3_ + H_2_O (4)

Consequently, highly concentrated NaOH solutions can absorb a conspicuous amount of CO_2_ while keeping PCO_2_ almost down to zero although the elevated pH of the solution causes safety concerns.

The aim of the present proof-of-principle study was to evaluate in-vitro the feasibility and the CO_2_ transfer efficacy of membrane lung ventilation with a NaOH solution. This type of ventilation was named “alkaline liquid ventilation”.

Different concentrations of NaOH were tested and the efficacy and efficiency in CO_2_ removal of alkaline liquid ventilation were compared to conventional sweep gas flow.

## 2. Materials and Methods

**Figure 1:**
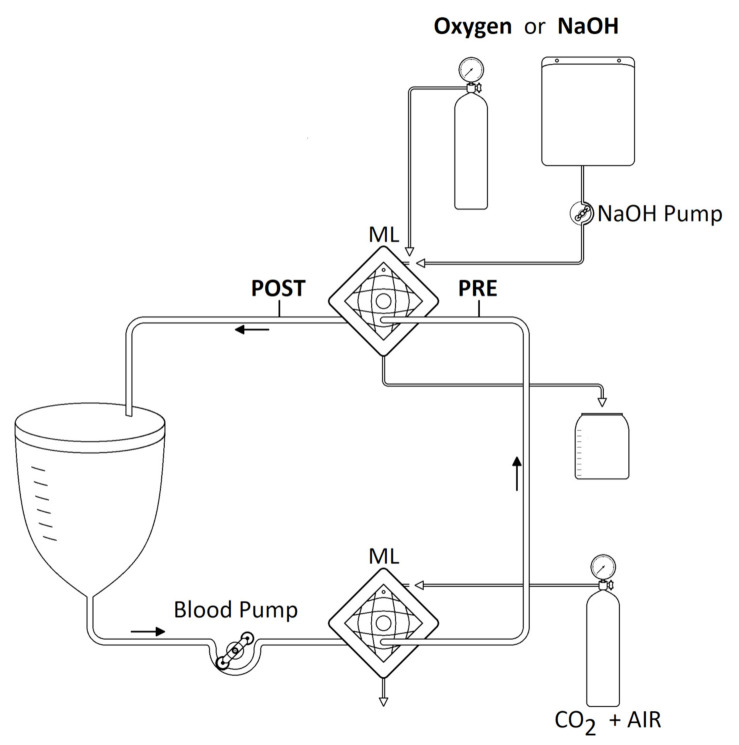
Schematic representation of the extracorporeal circuit. ML: membrane lung; PRE: blood sampling access upstream the ML for ECCO_2_R; POST: blood sampling access downstream the ML for ECCO_2_R.

An in vitro setting (Figure 1) was built to simulate a patient undergoing extracorporeal CO_2_ removal.

A closed-loop circuit was assembled with 3/8 and 1/4 inch polyvinylchloride class IV medical tubes (Medtronic, Minneapolis, MN, USA), one 4 L reservoir (VHK 71000 venous hardshell cardiotomy reservoir, Getinge, Gothenburg, Sweden), two polypropylene oxygenators membrane gas exchangers (Quadrox-i Small adult HMO 50000, Getinge, Gothenburg, Sweden) and one peristaltic pump (Multiflow Roller Pump Module H10 series, Stöckert Shiley, München, Germany).

The circuit was primed with about 3 L of swine blood collected at a local abattoir during usual slaughtering processes in compliance with CE regulations (1069/2009), authorization number 0141051/19 provided by ATS Milano, Regione Lombardia. MultiBic^®^ solution (Fresenius Medical Care Italia, Palazzo Pignano, Italy) was added to achieve a total volume of about 4 L. Sodium Heparin 25000 I.U. (Pfizer Italia S.r.l, Latina, Italy), anticoagulant-citrate-dextrose ACD 300 mL (Fresenius Kabi Italia, Isola Della Scala, Italy) and cefazolin 1 g (Teva Italia, Milano, Italy) were added to the blood.

The first gas exchanger downstream the reservoir was ventilated with a gas mixture of air and CO_2_ to maintain a constant PCO_2_ of 60 ± 2 mmHg at the inlet of the second oxygenator throughout all experiments. The second oxygenator was employed to remove CO_2_ through either ventilation with oxygen or a continuous infusion of sodium hydroxide (NaOH) solution, “alkaline liquid ventilation”, into the gas side of the membrane lung. Circuit accesses for blood sampling were positioned upstream (PRE) and downstream (POST) of the second oxygenator.

NaOH pellets (Sigma-Aldrich, Merck KGaA, Saint Louis, MO, USA) were diluted in distilled water to achieve the required concentrations. NaOH solutions were stored in disposable parenteral bags (Bertoni Nello S.r.l. Modena, Italy) and infused into the gas inlet port in the gas exchanger using a peristaltic pump (Multiflow Roller Pump Module H10 series, Stöckert Shiley, München, Germany). NaOH exiting the oxygenator was discarded.

The blood temperature was kept stable at 37 °C through heat exchangers connected to the membrane lungs.

The study was divided into four steps: (1) a mathematical modeling of NaOH and CO_2_ interactions to evaluate the theoretical basis of the study; (2) a safety and feasibility test to evaluate the effects of increasing NaOH concentrations on the membrane lung integrity and CO_2_ removal; (3) an efficiency test to compare the CO_2_ removal of similar sweep flows (up to 1 L × min^−1^) of oxygen vs. NaOH at the concentration selected following the feasibility test; (4) an efficacy test to compare the CO_2_ removal of the best liquid ventilation flow, selected from the efficiency test, vs. 10 L × min^−1^ of oxygen.

All the in-vitro tests were performed with 500 mL × min^−1^ of blood flow.

### 2.1. Mathematical Modeling

Theoretical effects of CO_2_ absorption by aqueous NaOH were computed solving a system of equations (MATLAB R2018b; The Math Works, Inc, Natick, MA, USA), including standard mass-action, mass-conservation and electroneutrality laws of the involved species: water, NaOH, CO_2_ (see the Online Supplement for more details).

We simulated a closed system with aqueous NaOH at varying concentrations (from 0 to 100 by 20 mmol × L^−1^) in which we introduced CO_2_ at different concentrations (from 0 to 100 by 5 mmol × L^−1^).

Of note, in the present mathematical model of a closed system, total pressure could exceed barometric pressure.

### 2.2. Definitions and Calculations

Bicarbonate ion concentration ([HCO_3_^−^]) was calculated from pH and PCO_2_ modifying the Henderson-Hasselbalch equation

(5) $$[{\mathrm{HCO}}_{3}^{-}]=\;\alpha \;\times {\;\mathrm{PCO}}_{2}\times 10^{\mathrm{pH}-\mathrm{pK}}$$

where α = 0.0307 mmol × L^−1^ × mmHg^−1^ (solubility of CO_2_ in plasma) [30,31] and pK = 6.129 (negative logarithm of the equilibrium constant) [31,32,33].

Plasma carbon dioxide content PRE and POST membrane lung (expressed in mmol × L^−1^) was calculated according to the method published by Douglas et al. [34]:

(6) $$[{\mathrm{TCO}}_{2}]=\;\alpha \;\times {\;\mathrm{PCO}}_{2}\times \left(1+10^{\mathrm{pH}-\mathrm{pK}}\right)$$

Carbon dioxide transfer across the membrane lung, $\dot{V}$CO_2_ (in mL × min^−1^), was calculated from the transmembrane lung TCO_2_ difference [35]:

(7) $${\dot{V}\mathrm{CO}}_{2}=(\left[{\mathrm{TCO}}_{2\mathrm{PRE}}]-[{\mathrm{TCO}}_{2\mathrm{POST}}]\right)\times \;\mathrm{blood}\;\mathrm{flow}\;\times 25.45$$

where TCO_2PRE_ represents CO_2_ content before the membrane lung while TCO_2POST_ is the CO_2_ content after the membrane lung, blood flow is measured in L × min^−1^, and the conversion factor is in mL × mmol^−1^.

### 2.3. Safety and Feasibility Test

Possible macroscopic detrimental effects on the membrane lung were evaluated. The effect on CO_2_ removal of alkaline liquid ventilation at increasing concentrations of NaOH (10, 30, 60, 90, 100 mmol × L^−1^) and increasing ventilating flows (100, 250, 500, 1000 mL × min^−1^) was likewise evaluated. Each combination of NaOH concentration and sweep fluid flow was tested once and for 15 min. At the end of each step, PRE and POST blood samples were collected for blood gas analysis (BGA) (Radiometer abl800 flex, Copenhagen, Denmark).

In addition, the integrity of the oxygenator was evaluated through visual inspection of the membrane lung, evaluation of the presence of blood in the NaOH solution exiting the oxygenator, and through analysis of blood sodium, potassium, and methemoglobin as indirect markers of hemolysis. The time-course of methemoglobin was evaluated at 4 time points (15, 30, 45, and 60 min) while testing aqueous NaOH at different sweep flows during the efficiency and efficacy tests.

The CO_2_ removal efficiency was estimated by computing PCO_2_ differences across the membrane lung and $\dot{V}$CO_2_.

At the end of the feasibility test, we selected the highest NaOH concentration endured by the membrane lung to perform the subsequent efficiency and efficacy tests.

### 2.4. Efficiency and Efficacy Tests

We performed six experiments with blood from 4 pigs. For each experiment, we tested, in random order, two different sweeping fluids, pure oxygen (FiO_2_ equal to 1) and aqueous NaOH at 100 mmol × L^−1^ (the concentration selected from the feasibility test). Four sweep flows (100, 250, 500, 1000 mL × min^−1^) for each fluid were randomly tested. We also randomized and tested 10 L/min of oxygen flow. Each combination of sweep fluid and flow was applied once during the single experiment.

The target PRE PCO_2_ was 60 ± 2 mmHg.

At the end of each step lasting about 15 min, we collected PRE and POST blood samples for BGA.

CO_2_ removal efficiency and efficacy were evaluated from PCO_2_ differences across the membrane lung and $\dot{V}$CO_2_.

The highest CO_2_ removal achieved with alkaline liquid ventilation was compared with the CO_2_ removal achieved with conventional gaseous ventilation performed with 10 L × min^−1^ of oxygen.

### 2.5. Statistical Analysis

Data are reported as median and interquartile range (IQR). Two-way repeated measures ANOVA or two-way repeated measures ANOVA on ranks was used, as appropriate, to test safety, feasibility (PRE and POST values), and efficiency.

One-way repeated measures or Friedman repeated measures was used, as appropriate, to test safety and feasibility (POST–PRE differences) and to compare methemoglobin values at different time points.

Paired *t*-test or Wilcoxon signed rank test was used, as appropriate, to test efficacy. Post-hoc analyses were performed with Bonferroni or Tukey corrections. Statistical significance was defined as *p* < 0.05. Analysis was performed with SAS software 9.4 (SAS Institute, Inc., Cary, NC, USA) and SigmaPlot v.11.0 (Systat Software Inc, San Jose, CA, USA).

## 3. Results

### 3.1. Mathematical Modeling

**Figure 2:**
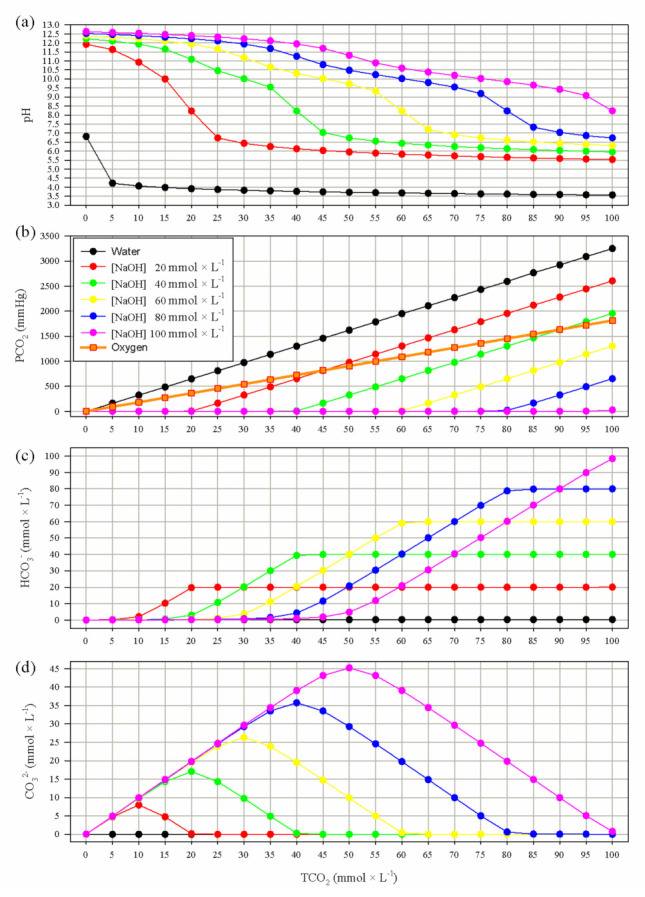
Simulated effects of increasing TCO_2_ from 0 to 100 by 5 mmol × L^−1^ in a *closed* system with aqueous NaOH at varying concentrations (from 0 (water) to 100 by 20 mmol × L^−1^). Panel (**a**) represents pH; panel (**b**) represents PCO_2_, the orange line with red squares represents PCO_2_ values of one *closed* liter of oxygen/gas containing increasing TCO_2_; panel (**c**) represents HCO_3_^−^; panel (**d**) represents CO_3_^2−^. Abbreviations: PCO_2_, partial pressure of carbon dioxide; HCO_3_^−^, bicarbonate; CO_3_^2−^, carbonate; TCO_2_, total CO_2_ content.

The PCO_2_ of gas/oxygen or distilled water, in a closed system, at increasing concentrations of CO_2_ raises linearly, see Figure 2, although the slope is steeper in water relative to gas/oxygen. Instead, if NaOH is added to water, the solution PCO_2_ remains close to zero as long as the added CO_2_ is lower than the amount of added NaOH. When similar amounts of CO_2_ and NaOH are added, almost all CO_2_ reacts forming HCO_3_^−^ and the solution pH is around 8.220–8.230.

Otherwise, if the added CO_2_ is lower than NaOH, carbonic acid dissociates to HCO_3_^−^ which, due to the alkaline milieu, further dissociates to CO_3_^2−^, thus reducing the concentration of HCO_3_^−^. When CO_2_ is near half or lower than NaOH, almost all CO_2_ forms CO_3_^2−^ and the solution pH is above 11. Instead, if the TCO_2_ is higher than NaOH, all hydroxide reacts with CO_2_ forming HCO_3_^−^ and the pH decreases below 8.

Interestingly when the added CO_2_ is higher than twice the NaOH, the PCO_2_ in the NaOH solution will be higher than the one in a similar gas volume containing the same amount of CO_2_.

For example, one liter of gas containing 200 mL (7.86 mmol) of CO_2_, the theoretical $\dot{V}$CO_2_ of an adult, would have a PCO_2_ of 143 mmHg (713 mmHg × 0.2), while 1 L of water would have a higher PCO_2_ of 255 mmHg. On the contrary, the same amount of CO_2_ could be stored in 1 L of NaOH 10 mmol × min^−1^ solution with a PCO_2_ close to zero.

### 3.2. Feasibility and Safety Test

No detectable damages to the membrane lung were observed. Moreover, no blood was found in the sweep fluid exiting the oxygenator.

**Figure 3:**
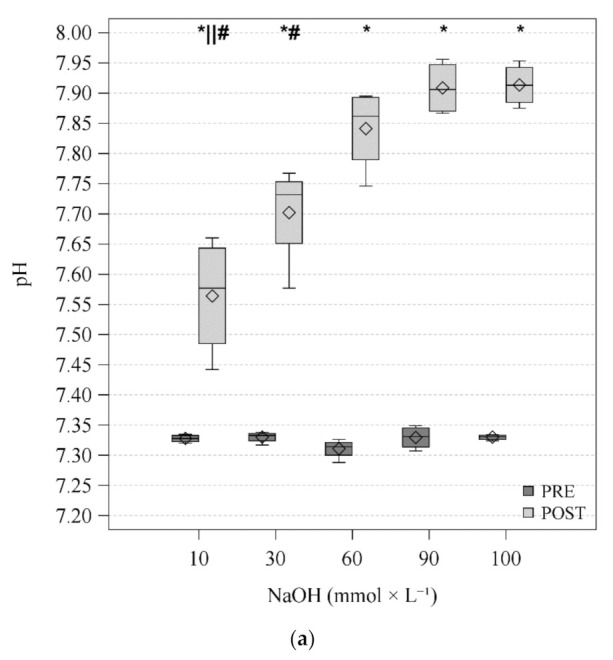

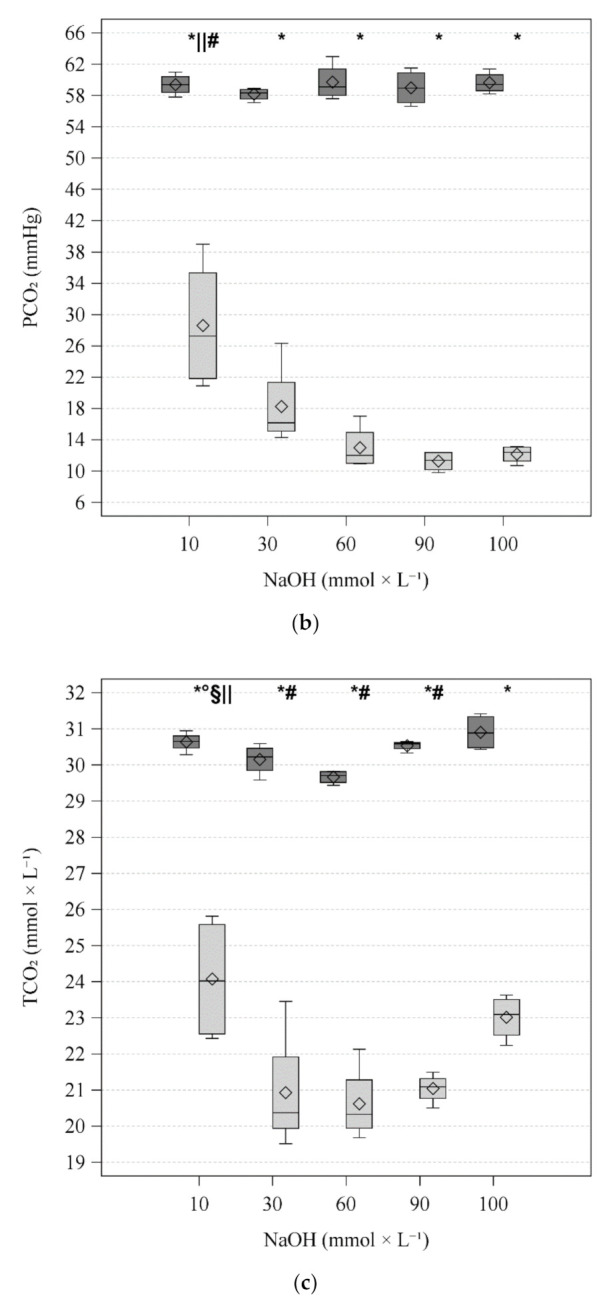
Figures display the distribution of data by using a rectangular box plot and whiskers, the bottom and top edges of the box indicate the intra-quartile range (IQR) between the first and third quartiles (the 25th and 75th percentiles). The diamond marker inside the box indicates the mean value. The line inside the box indicates the median value. Whiskers indicate the range of values outside of the intra-quartile range but at a distance lower than the upper and lower fences (±1.5 × IQR). Dark grey represents PRE blood sampling. Light grey represents POST blood sampling. Statistical analysis: Two-way ANOVA RM (TCO_2_) or two-way ANOVA RM on ranks (pH and PCO_2_). * *p* < 0.05 vs. PRE; ° *p* < 0.05 vs. 30; § *p* < 0.05 vs. 60; || *p* < 0.05 vs. 90; # *p* < 0.05 vs. 100. (**a**) pH distribution according at different NaOH concentrations; (**b**) PCO_2_ (partial pressure of carbon dioxide) distribution at different NaOH concentrations. (**c**) TCO_2_ (Carbon dioxide content) distribution at different NaOH concentrations.

Figure 3 and Table 1 report the BGAs of PRE and POST blood. PCO_2PRE_ was stable throughout the entire test, 59.0 (58.0–60.0) mmHg. Delta PCO_2_ across the membrane lung was significantly lower at 10 mmol × L^−1^ (−32.2 (−38.6–−23.1) mmHg). Otherwise, it showed small increases as NaOH concentration increases (−41.4 (−43.1–−36.8), −47.7 (−49.5–−44), −47.8 (−48.6–−47) and −48.2 (−48.4–−46.6) mmHg at 30, 60, 90, and 100 mmol × L^−1^ respectively). PCO_2POST_ was reduced to about 12 mmHg with NaOH concentration ≥ 60 mmol × L^−1^ (12.0 (11.0–15.0), 11.4 (10.2–12.4) and 12.4 (11.3–13.1) mmHg at 60, 90, and 100 mmol × L^−1^ respectively), subsequently pH_POST_ increased up to 7.913 (7.885–7.943) at NaOH concentration equal to 100 mmol × L^−1^. The lowest $\dot{V}$CO_2_ was also recorded at the lowest NaOH concentration 73.9 (54.3–91.8) mL × min^−1^. PRE blood sodium and potassium concentration were stable (see Supplementary Table S1 for details). POST chloride concentrations were higher than PRE values while sodium concentrations were lower. Moreover, a simultaneous decrease in potassium and calcium POST concentrations was observed. These results are similar to the observations of Langer et al. in couples of measurements of blood entering and leaving the ML in 20 critically ill patients [36]. Methemoglobin values were not different over the time during the experiments (median (IQR) values 1.100 (0.950–2.850) at 15 min, 1.100 (1.050–2.150) at 30 min, 1.100 (1.050–2.400) at 45 min, 1.200 (1.050–2.900) at 60 min; *p* = 0.606).

As the highest delta PCO_2_ was observed when 100 mmol × L^−1^ NaOH was used, this concentration was employed for the efficiency and efficacy tests.

### 3.3. Efficiency Test

**Figure 4:**
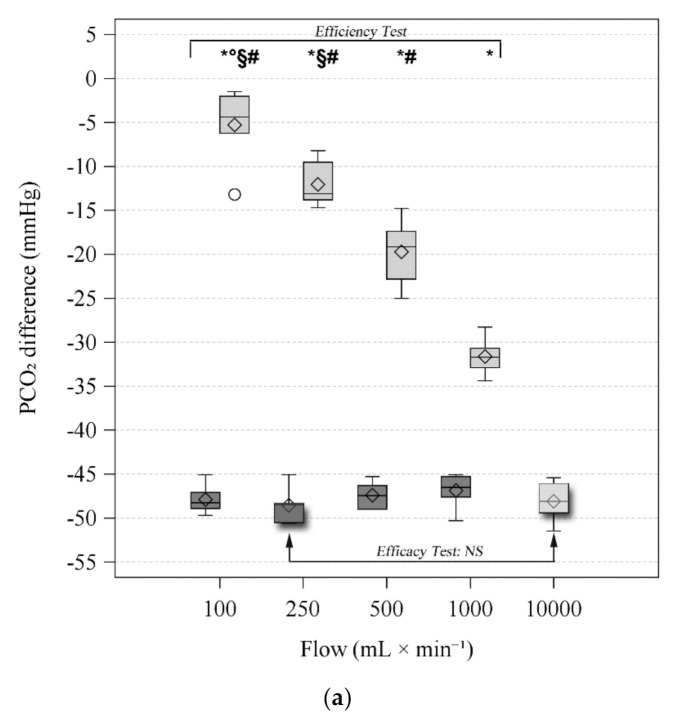

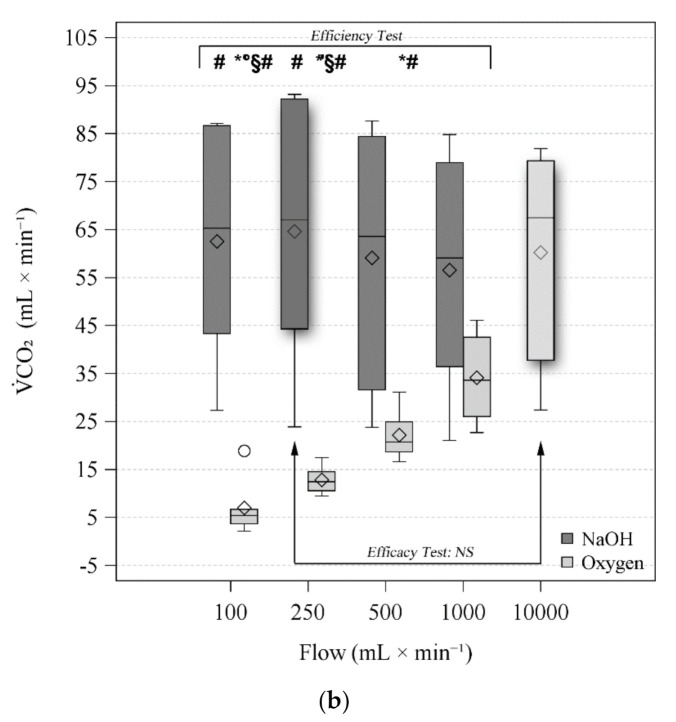
Figures display the distribution of data by using a rectangular box plot and whiskers, the bottom and top edges of the box indicate the intra-quartile range (IQR) between the first and third quartiles (the 25th and 75th percentiles). The diamond marker inside the box indicates the mean value. The line inside the box indicates the median value. Whiskers indicate the range of values outside of the intra-quartile range but at a distance lower than the upper and lower fences (±1.5 × IQR) Dots represent outliers (observations that are more extreme than the upper and lower fences). Dark grey represents NaOH at 100 mmol × L^−1^ concentration. Light grey represents Oxygen. Efficiency test statistical analysis: Two-way ANOVA RM. * *p* < 0.05 vs. NaOH; ° *p* < 0.05 vs. 250 mL × min^−1^; § *p* < 0.05 vs. 500 mL × min^−1^; # *p* < 0.05 vs. 1000 mL × min^−1^. Efficacy test statistical analysis: Paired t-test between NaOH at 100 mmol × L^−1^ concentration and 250 mL × min^−1^ sweep flow and oxygen at 1000 mL × min^−1^ sweep flow (boxes highlighted by outside shadow and arrows). (**a**) PCO_2_ (partial pressure of carbon dioxide) difference (POST values–PRE values) distribution according to different sweep flows of NaOH and Oxygen. (**b**) $\dot{V}$CO_2_ (Carbon dioxide transfer across the membrane lung) distribution according to different sweep flows of NaOH and Oxygen.

Blood gas analyses of PRE and POST blood with NaOH and oxygen are reported in Table 1. PCO_2PRE_ was stable throughout the entire test, 59.6 (58.9–60.4) mmHg. Increasing oxygen flows showed increasing CO_2_ removal, both as delta PCO_2_ across the membrane lung and $\dot{V}$CO_2_, see Figure 4. Conversely, all NaOH flows showed similar CO_2_ removal, except for a lower $\dot{V}$CO_2_ at 1000 mL × min^−1^ compared to 100 and 250 mL × min^−1^ (see Figure 3). When comparing $\dot{V}$CO_2_ achieved with liquid and gaseous ventilation, liquid ventilation achieved significantly higher CO_2_ removals for 100, 250, and 500 mL × min^−1^ of flow. On the contrary, while the median value was higher also for 1000 mL × min^−1^, this difference did not reach statistical significance.

Blood pH_POST_ increased, according to the PCO_2_ reduction, reaching values as high as 7.987 with NaOH at 250 mL × min^−1^.

PO_2PRE_ was stable around 141.0 (137.0–147.0) mmHg both during NaOH and oxygen steps while PO_2POST_ increased up to 470.5 (452.3–507.0) mmHg only during oxygenation use, while it remained unchanged during liquid ventilation.

Blood electrolytes and lactate concentrations were stable throughout the experiment.

### 3.4. Efficacy Tests

In agreement with the highest $\dot{V}$CO_2_ and delta PCO_2_, NaOH 250 mL × min^−1^ was selected as the most performant NaOH flow and compared with 10 L × min^−1^ of oxygen in the efficacy test. Table 2 reports blood gas analyses of PRE and POST blood. Both delta PCO_2_ and $\dot{V}$CO_2_ were similar, suggesting similar extracorporeal CO_2_ removal (Figure 4, shadowed boxes).

## 4. Discussion

This in-vitro study shows that continuous infusion (from 100 to 1000 mL × min^−1^) of highly concentrated sodium hydroxide solutions into the gas side of conventional polypropylene oxygenators is feasible, despite pH values of the sweeping solution above 12. At low sweep flows, alkaline liquid ventilation showed significantly higher CO_2_ removal capacity than conventional gaseous ventilation. However, the maximum CO_2_ removal efficiency achieved through liquid ventilation was not superior to the one achieved with 10 L × min^−1^ of sweep gas flow.

The working hypothesis underlying this study was to exploit the high CO_2_ absorbing capacity of NaOH solutions. Indeed, in our experimental context, the concentration of NaOH was always significantly higher than the amount of CO_2_ extracted from the ML. The PCO_2_ of the alkaline sweep fluid was persistently very close to 0 mmHg, as the added CO_2_ was instantly hydrated and dissociated to bicarbonate and carbonate. This allowed to keep the PCO_2_ close to zero and thus optimize the transmembrane PCO_2_ gradient, favoring the efficiency of extracorporeal CO_2_ removal.

Indeed, a solution containing 10 mEq of NaOH could absorb 200 mL of CO_2_ while maintaining PCO_2_ close to zero. On the contrary, the same amount of CO_2_ added to 10 L of gas would result in a PCO_2_ around 15 mmHg, therefore reducing the blood-gas CO_2_ gradient.

However, the data showed that increasing NaOH flow did not lead to a linear increase in CO_2_ removal. Instead, for NaOH flows greater than 250 mL × min^−^ there was an unexpected reduction in CO_2_ removal. This reduced efficiency could depend on the density of the sodium hydroxide solution and the mechanics of the membrane lung. Therefore, a clinical application of alkaline liquid ventilation does not seem exploitable using the current technology. The technical complexity and safety profile require further evaluations, although the present tests have recorded no damage to the membrane lung.

Another important difference between gaseous and liquid ventilation needs to be discussed. Although the oxygenation capacity of low-flow devices using conventional gaseous ventilation is limited by the amount of blood reaching the ML, a certain amount of oxygen is added to the blood. On the contrary, the NaOH infusion does not oxygenate the extracorporeal blood, limiting its potential clinical application to patients with isolated hypercapnic respiratory failure, i.e., able to oxygenate properly through their native lungs.

Although devices with higher CO_2_ extraction capacity resulted more effective [3,9,10], numerous studies confirm the ability of ECCO_2_R to achieve physiological targets. Nevertheless, the clinical application of ECCO_2_R is still limited and no conclusive indications have been identified mainly because of safety concerns [37,38]. Indeed, a consistently high rate of complications has been reported, mostly related to hemolysis, bleeding, and thrombosis. In this context, the present study aim was to achieve a highly efficient ECCO_2_R technique to ensure a clinical efficacy with limited extracorporeal blood flows, potentially enabling regional anticoagulation [39,40]. The tested technology, which was not developed for alkaline liquid ventilation, did not meet such expectations. However, we cannot exclude that a dedicated device could achieve more satisfying results.

This study presents several limitations. First, we could not perform any gas analysis of the sodium hydroxide solution. The CO_2_ extraction capacity was estimated both as differences in PCO_2_ and TCO_2_ between the blood samples upstream and downstream of the artificial lung [41]. $\dot{V}$CO_2_ showed higher variability than PCO_2_, as shown in Figure 4, possibly due to the baseline different blood composition. Indeed, we can speculate that this phenomenon may be explained by the wide range in hemoglobin concentrations (see Table 1), which affects the ML $\dot{V}$CO_2_ [42]. Secondly, the alkaline liquid ventilation was tested only in vitro and for a limited time consequently we cannot exclude different effects and safety issues in vivo scenarios. Thirdly, we only tested one type of polypropylene membrane lung. Further tests with different devices may be required.

## 5. Conclusions

This in-vitro study showed that ECCO_2_R through alkaline liquid ventilation of the ML is feasible and safe. The CO_2_ removal efficiency of alkaline liquid ventilation was higher than conventional gaseous ML ventilation only for low sweep flows. Indeed, at high sweep gas flow, the CO_2_ removal efficiency was comparable between the two techniques.

The development of a dedicated device may be necessary to exploit the potential of this technology. Further studies will be required before any possible clinical application.

## Figures and Tables

**Table 1:** Efficiency tests results.

Variable		Ventilation	Flow (L × min^−1^)				*p* Vent.	*p* Flow	*p* Int.
			100	250	500	1000			
**pH**	**PRE ^$^**	NaOH	7.346 (7.337–7.374)	7.351 (7.333–7.359)	7.356 (7.333–7.363)	7.336 (7.334–7.366)	**0.027**	0.999	**0.020**
		O_2_	7.325 (7.306–7.333) *	7.313 (7.311–7.349) *	7.321 (7.301–7.34) *	7.325 (7.318–7.346)			
	**POST ^$^**	NaOH	7.972 (7.968–8.057) #	7.987 (7.977–8.077) §#	7.964 (7.932–8.040)	7.938 (7.902–8.008)	**<0.001**	**<0.001**	**<0.001**
		O_2_	7.352 (7.333–7.379) *°§#	7.405 (7.374–7.439) *§#	7.481 (7.435–7.514) *#	7.616 (7.612–7.654) *			
	**Difference ^$^**	NaOH	0.628 (0.597–0.683) #	0.643 (0.624–0.718) §#	0.606 (0.597–0.673)	0.591 (0.565–0.635)	**<0.001**	**<0.001**	**<0.001**
		O_2_	0.028 (0.011–0.041) *°§#	0.094 (0.063–0.101) *§#	0.145 (0.124–0.186) *#	0.295 (0.268–0.326) *			
**PCO_2_** **(mmHg)**	**PRE**	NaOH	59.7 (59.2–60.1)	59.5 (59.0–59.7)	59.4 (58.4–60.2)	60.0 (59.2–60.4)	0.909	0.882	0.332
		O_2_	59.0 (58.4–59.9)	59.0 (58.7–60.5)	60.6 (59.0–61.0)	59.7 (59.5–59.8)			
	**POST**	NaOH	11.2 (11.0–13.0)	10.5 (10.3–11.1)	11.7 (11.3–12.1)	13.1 (12.8–13.1)	**<0.001**	**<0.001**	**<0.001**
		O_2_	54.6 (53.7–56.2) *°§#	46.2 (45.4–49.7) *§#	40.4 (39.0–41.6) *#	28.2 (26.9–29.1) *			
	**Difference**	NaOH	−48.3 (−48.9–−47.1)	−48.5 (−50.5–−48.3)	−47.5 (−49.0–−46.3)	−46.5 (−47.6–−45.3)	**<0.001**	**<0.001**	**<0.001**
		O_2_	−4.4 (−6.2–−2.0) *°§#	−13.1 (−13.8–−9.5) *§#	−19.1 (−22.8–−17.4) *#	−31.7 (−32.9–−30.7) *			
**PO_2_** **(mmHg)**	**PRE**	NaOH	138.0 (136.0–139.0) #	137.0 (137.0–143.0) #	137.5 (136.0–146.0)	141.5 (137.0–153.0)	0.231	0.460	**0.002**
		O_2_	144.0 (141.0–162.0)	143.0 (140.0–159.0)	143.5 (138.0–156.0)	143.0 (139.0–154.0)			
	**POST ^$^**	NaOH	125.0 (120.0–130.0)§#	130.5 (128.0–140.0) #	148.5 (142.0–157.0)	161.5 (159.0–169.0)	**<0.001**	**<0.001**	0.700
		O_2_	595.5 (591.0–602.0) *§#	608.5 (603.0–623.0) *#	616.0 (611.0.–6260) *#	648.0 (632.0–654.0) *			
	**Difference**	NaOH	−13.0 (−16.0— −11.0)§#	−6.5 (−9.0–−3.0) §#	9.0 (6.0–11.0) #	18.0 (14.0–21.0)	**<0.001**	**<0.001**	0.105
		O_2_	451.5 (429.0–461.0) *§#	455.5 (445.0–471.0) *§#	462.5 (453.0–477.0) *#	497.5 (487.0–508.0) *			
**K^+^** **(mEq × L^−1^)**	**PRE**	NaOH	4.1 (4.0–4.4)	4.1 (4.1–4.5)	4.2 (4.1–4.4)	4.2 (4.1–4.5)	0.127	0.594	0.299
		O_2_	4.1 (3.9–4.2)	4.0 (4.0–4.2)	4.0 (4.0–4.2)	4.1 (4.0–4.2)			
	**POST**	NaOH	4.1 (4.0–4.3)	4.1 (4.0–4.4)	4.1 (4.0–4.3)	4.1 (4.0–4.4)	0.265	0.709	0.363
		O_2_	4.1 (3.9–4.2)	4.0 (4.0–4.2)	4.0 (4.0–4.1)	4.0 (3.9–4.2)			
	**Difference**	NaOH	0.0 (0.0–0.1)	0.1 (0.0–0.1)	0.1 (0.1–0.1)	0.1 (0.1–0.1)	**0.009**	0.337	0.86
		O_2_	0.0 (0.0–0.0) *	0.0 (0.0–0.0) *	0.0 (0.0–0.0) *	0.0 (0.0–0.1) *			
**Na^+^** **(mEq × L^−1^)**	**PRE**	NaOH	143.0 (142.0–144.0)	143.0 (143.0–144.0)	143.5 (142.0–145.0)	143.5 (143.0–144.0)	**0.038**	0.233	0.973
		O_2_	139.0 (138.0–143.0) *	139.0 (139.0–143.0) *	139.5 (138.0–144.0) *	139.5 (138.0–145.0) *			
	**POST**	NaOH	141.0 (139.0–142.0)	140.0 (140.0–142.0)	140.5 (140.0–141.0)	141.5 (140.0–142.0)	0.407	0.524	0.096
		O_2_	139.0 (138.0–143.0)	138.5 (137.0–143.0)	139.0 (138.0–143.0)	138.5 (137.0–143.0)			
	**Difference**	NaOH	−2.0 (−3.0–−2.0)	−3.0 (−3.0–−2.0)	−3.0 (−4.0–−2.0)	−2.0 (−3.0–−2.0)	**<0.001**	0.215	**0.012**
		O_2_	0.0 (0.0–0.0) *#	−1.0 (−1.0–0.0) *	−1.0 (−1.0–−1.0) *	−1.0 (−1.0–−1.0) *			
**Ca^++^** **(mEq × L^−1^)**	**PRE**	NaOH	1.3 (1.3–1.4)	1.4 (1.3–1.4)	1.4 (1.3–1.4)	1.4 (1.3–1.4)	0.755	0.854	0.769
		O_2_	1.4 (1.2–1.4)	1.3 (1.2–1.4)	1.3 (1.2–1.4)	1.3 (1.2–1.4)			
	**POST**	NaOH	1.2 (1.1–1.3)	1.2 (1.1–1.2)	1.2 (1.2–1.3)	1.2 (1.2–1.3)	**0.066**	0.110	**<0.001**
		O_2_	1.4 (1.2–1.4) *§#	1.3 (1.2–1.4) *§#	1.3 (1.2–1.3)	1.3 (1.2–1.3)			
	**Difference ^$^**	NaOH	−0.1 (−0.1–−0.1)	−0.2 (−0.2–−0.1)	−0.1 (−0.1–−0.1)	−0.1 (−0.1–−0.1)	**<0.001**	**0.032**	**0.002**
		O_2_	0.0 (0.0–0.0) *§#	0.0 (0.0–0.0) *§#	0.0 (0.0–0.0) *	−0.1 (−0.1–−0.1) *			
**Cl^−^** **(mEq × L^−1^)**	**PRE**	NaOH	111.5 (111.0–113.0)	111.5 (111.0–113.0)	111.5 (111.0–113.0)	111.5 (110.0–113.0)	0.232	0.529	0.529
		O_2_	111.0 (111.0–112.0)	111.0 (111.0–112.0)	111.0 (110.0–112.0)	111.0 (110.0–112.0)			
	**POST**	NaOH	114.0 (114.0–115.0)	114.5 (114.0–115.0)	114.0 (114.0–115.0)	114.0 (114.0–115.0)	**0.002**	**0.042**	**0.002**
		O_2_	111.5 (111.0–113.0) *#	111.0 (111.0–113.0) *#	112.0 (111.0— 113.0) *#	112.5 (112.0–114.0) *			
	**Difference**	NaOH	2.5 (2.0–3.0)	3.0 (2.0–3.0)	2.5 (2.0–3.0)	2.5 (2.0–3.0)	**0.007**	**0.002**	**0.001**
		O_2_	0.5 (0.0–1.0) *#	0.0 (0.0–1.0) *§#	1.0 (1.0–1.0) *#	2.0 (1.0–2.0)			
**Lac** **(mEq × L^−1^)**	**PRE ^$^**	NaOH	1.4 (0.5–2.3)	1.4 (0.5–2.4)	1.3 (0.5–2.3)	1.4 (0.5–2.5)	0.180	0.361	0.614
		O_2_	1.1 (0.4–2.5)	1.2 (0.4–2.6)	1.1 (0.4–2.6)	1.1 (0.4–2.5)			
	**POST ^$^**	NaOH	1.4 (0.5–2.3)	1.5 (0.5–2.3)	1.4 (0.5–2.4)	1.4 (0.5–2.4)	0.197	0.459	0.850
		O_2_	1.0 (0.4–2.6)	1.1 (0.4–2.6)	1.2 (0.4–2.6)	1.1 (0.4–2.5)			
	**Difference**	NaOH	0.0 (0.0–0.0)	−0.1 (−0.1–0.1)	0.1 (0.0–0.1)	0.0 (0.0–0.0)	1.000	0.297	0.922
		O_2_	0.0 (0.0–0.0)	0.0 (−0.1–0.0)	0.0 (0.0–0.1)	0.0 (0.0–0.0)			
**Hb** **(g × dL^−1^)**	**PRE**	NaOH	6.45 (5.50–8.20)	6.80 (5.30–8.20)	6.70 (5.30–8.30)	6.70 (5.20–8.10)	0.643	0.641	0.511
		O_2_	6.55 (5.50–8.30)	6.60 (5.30–8.20)	6.55 (5.30–8.40)	6.55 (5.40–7.90)			
	**POST**	NaOH	6.60 (5.50–8.30)	6.70 (5.40–8.20)	6.75(5.40–8.30)	6.60 (5.30–8.20)	0.547	0.083	0.893
		O_2_	6.55 (5.50–8.40)	6.60 (5.30–8.20)	6.60 (5.30–8.50)	6.55 (5.40–7.90)			
	**Difference**	NaOH	0.05 (0.00–0.10)	0 (−0.10–0.00)	0.05 (0.00–0.10)	0.05 (0.00–0.10)	**0.025**	0.661	0.154
		O_2_	0.00 (−0.10–0.00) *	0.00 (0.00–0.00) *	0.00 (0.00–0.10) *	0.00 (0.00–0.00) *			
**HCO_3_^−^** **(mmol × L^−1^)**	**PRE**	NaOH	30.1 (29.5–32.2)	30.2 (29.3–31.8)	30.3 (29.4–31.7)	29.8 (29.0–31.8)	0.050	0.508	0.165
		O_2_	28.3 (27.6–29.9) *	28.2 (27.6–29.8) *	28.1 (27.5–30.4) *	28.5 (28.2–30.2) *			
	**POST**	NaOH	25.7 (23.3–29.1)	25.9 (23.2–28.1)	26.4 (23.2–29)	26.3 (23.7–29.7)	0.577	**0.043**	**0.003**
		O_2_	28.0 (27.2–28.6) §#	27.5 (26.7–28.6) #	27.0 (26.3–28.2)	26.6 (26.1–27.2)			
	**Difference**	NaOH	−4.4 (−6.3–−2.4) #	−4.5 (−6.8–−2.4) #	−4.2 (−6.2–−1.3)	−3.9 (−5.7–−1.7)	**0.018**	**0.003**	**<0.001**
		O_2_	−0.3 (−0.5–−0.2) *§#	−0.8 (−0.9–−0.6) *§#	−1.3 (−1.7–−1) *#	−2.1 (−2.8–−1.4)			
**plasma TCO_2_** **(mmol × L^−1^)**	**PRE**	NaOH	31.9 (31.4–34.1)	32.0 (31.1–33.6)	32.1 (31.3–33.5)	31.7 (30.9–33.6)	0.051	0.476	0.194
		O_2_	30.1 (29.5–31.8)	30.1 (29.4–31.5)	30.0 (29.4–32.3)	30.3 (30.1–32.0)			
	**POST**	NaOH	26.0 (23.6–29.5)	26.3 (23.5–28.4)	26.7 (23.6–29.3)	26.7 (24.1–30.1)	0.258	**0.009**	**<0.001**
		O_2_	29.7 (28.9–30.1) §#	29.1 (28.1–30.0) #	28.3 (27.4–29.5) #	27.5 (26.9–28.1)			
	**Difference**	NaOH	−5.9 (−7.8–−3.9) #	−6 (−8.3–−4.0) #	−5.7 (−7.6–−2.8)	−5.3 (−7.1–−3.3)	**0.006**	**<0.001**	**<0.001**
		O_2_	−0.5 (−0.6–−0.3) *°§#	−1.1 (−1.3–−0.9) *§#	−1.9 (−2.2–−1.7) *#	−3.0 (−3.8–−2.3)			
**$\dot{V}$CO_2_** **(mL × min^−1^)**		NaOH	65.3 (43.3–86.7) #	67.0 (44.3–92.2) #	63.5 (31.6–84.5)	59.1 (36.4–79)	**0.006**	**<0.001**	**<0.001**
		O_2_	5.4 (3.7–6.7) *°§#	12.5 (10.5–14.6) *§#	20.7 (18.6–24.9) *#	33.6 (26.1–42.6)			

Abbreviations: PCO_2_, partial pressure of carbon dioxide; PO_2_, partial pressure of oxygen; Na^+^, sodium; K^+^, potassium; Ca^++^, calcium; Cl^−^, chloride; Lac, Lactate; Hb, hemoglobin; HCO_3_-, bicarbonate, TCO_2_, total CO_2_ content, $\dot{V}$CO_2_, amount of carbon dioxide removed by the membrane lung. Data are expressed median (IQR); Differences were computed as POST values–PRE values. *p*: *p* values of two-way ANOVA RM or two-way ANOVA RM on ranks (^$^) for NaOH vs. O_2_ comparison (*p* Ventilation), Flow effect (*p* Flow) and interaction (*p* int.); Post-hoc analysis with Bonferroni or Tukey corrections: * *p* < 0.05 vs. NaOH; ° *p* < 0.05 vs. 250 mL/min; § *p* < 0.05 vs. 500 mL/min; # *p* < 0.05 vs. 1000 mL × min^−1^.

**Table 2:** Efficacy tests results.

Variable		Ventilation		
		NaOH 250 mL × min^−1^	O_2_ 10,000 mL × min^−1^	*p*
**pH**	**PRE**	7.351 (7.333–7.359)	7.328 (7.322–7.355)	**0.032**
	**POST**	7.987 (7.977–8.077)	7.966 (7.921–8.013)	**0.020**
	**Difference**	0.643 (0.624–0.718)	0.627 (0.599–0.685)	0.094
**PCO_2_ (mmHg)**	**PRE**	59.5 (59–59.7)	60 (59.3–60.5)	0.254
	**POST**	10.5 (10.3–11.1)	11.5 (10.8–13.9)	0.106
	**Difference**	−48.5 (−50.5–−48.3)	−48.1 (−49.4–−46.1)	0.522
**PO_2_ (mmHg)**	**PRE ^$^**	137 (137–143)	139 (137–165)	0.625
	**POST**	130.5 (128–140)	661.5 (649–677)	**<0.001**
	**Difference**	−6.5 (−9–−3)	518.5 (509–536)	**<0.001**
**K^+^ (mEq × L^−1^)**	**PRE**	4.1 (4.1–4.5)	4.2 (4–4.4)	0.611
	**POST ^$^**	4.1 (4–4.4)	4.1 (4–4.3)	0.438
	**Difference**	0.1 (0–0.1)	0.1 (0–0.1)	1.000
**Na^+^ (mEq × L^−1^)**	**PRE**	143 (143–144)	142 (141–145)	0.516
	**POST**	140 (140–142)	139.5 (139–143)	1.000
	**Difference**	−3 (−3–−2)	−2 (−2–−2)	0.102
**Ca^++^ (mEq × L^−1^)**	**PRE**	1.4 (1.3–1.4)	1.3 (1.3–1.4)	0.927
	**POST**	1.2 (1.1–1.2)	1.2 (1.1–1.3)	0.413
	**Difference**	−0.2 (−0.2–−0.1)	−0.1 (−0.1–−0.1)	0.067
**Cl^−^ (mEq × L^−1^)**	**PRE ^$^**	111.5 (111–113)	111.5 (111–112)	0.813
	**POST**	114.5 (114–115)	115 (114–115)	1.000
	**Difference**	3 (2–3)	3 (2–3)	0.611
**Lac (mEq × L^−1^)**	**PRE ^$^**	1.4 (0.5–2.4)	1.3 (0.5–2.5)	0.375
	**POST**	1.5 (0.5–2.3)	1.2 (0.4–2.5)	0.233
	**Difference**	−0.1 (−0.1–0.1)	0 (−0.1–0)	1.000
**Hb (g × dL^−1^)**	**PRE**	6.80 (5.30–8.20)	6.55 (5.50–8.40)	0.499
	**POST**	6.70 (5.40–8.20)	6.55 (5.40–7.90)	0.590
	**Difference**	0.00 (−0.10–0.00)	0.00 (0.00–0.00)	0.363
**HCO_3_^−^ (mmol × L^−1^)**	**PRE**	30.2 (29.3–31.8)	29.4 (28.7–30.6)	0.205
	**POST**	25.9 (23.2–28.1)	25.1 (23.1–28.1)	0.652
	**Difference**	−4.5 (−6.8–−2.4)	−4.6 (−5.7–−1.8)	0.185
**plasma TCO_2_ (mmol × L^−1^)**	**PRE ^$^**	32 (31.1–33.6)	31.3 (30.6–32.5)	0.313
	**POST**	26.3 (23.5–28.4)	25.5 (23.5–28.4)	0.695
	**Difference**	−6 (−8.3–−4)	−6.1 (−7.1–−3.4)	0.191
**$\dot{V}$CO_2_ (mL × min^−1^)**		67 (44.3–92.2)	67.4 (37.8–79.4)	0.191

Abbreviations: PCO_2_, partial pressure of carbon dioxide; PO_2_, partial pressure of oxygen; Na^+^, sodium; K^+^, potassium; Ca^++^, calcium; Cl^−^, chloride; Lac, Lactate; Hb, hemoglobin; HCO_3_^−^, Bicarbonate, TCO_2_, total CO_2_ content, $\dot{V}$CO_2_, amount of carbon dioxide removed by the membrane lung. Data are expressed median (IQR); Differences were computed as POST values–PRE values. *p*: *p* values of Paired *t*-test or Wilcoxon Signed Rank Test (^$^) for NaOH (250 mL × min^−1^) vs. O_2_ (10,000 mL × min^−1^) comparison.

## Data Availability

The dataset used and/or analyzed during the current study are available from the corresponding author on reasonable request.

## Supplementary Materials

The following are available online at https://www.mdpi.com/article/10.3390/membranes11070464/s1, The Online Supplement: Alkaline_liquid_ventilation_online_supplement.docx.

## Abbreviations

ACD, anticoagulant-citrate-dextrose; ARDS acute respiratory distress syndrome; Ca^++^, calcium; Cl^−^, chloride; CO_2_, carbon dioxide; CO_3_^2−^, carbonate; COPD, chronic obstructive pulmonary disease; ECCO_2_R, extracorporeal carbon dioxide removal; H_2_CO_3_, carbonic acid; H_2_O, water; Hb, hemoglobin; HCO_3_^−^, bicarbonate; IQR, interquartile range; K^+^, potassium; Lac, Lactate; ML, membrane lung; Na^+^, sodium; Na_2_CO_3_, sodium carbonate; NaHCO3, sodium bicarbonate; NaOH, sodium hydroxide; PaCO_2_, arterial partial pressure of CO_2_; PCO_2_, partial pressure of carbon dioxide; PO_2_, partial pressure of oxygen; TCO_2_, total CO_2_ content; $\dot{V}$CO_2_, amount of carbon dioxide removed by the membrane lung in one minute.
