# Supplementary material for: Alkaline Liquid Ventilation of the Membrane Lung for Extracorporeal Carbon Dioxide Removal (ECCO2R): In Vitro Study
Source: Membranes (Basel). 2021 Jun 22;11(7):464. doi: 10.3390/membranes11070464 (PMC8306443; doi:10.3390/membranes11070464)
Supplement: Supplementary file 1 [file membranes-11-00464-s001.zip › membranes-1258798-supplementary.pdf]

membranes

Supplementary Information

Alkaline Liquid Ventilation of the Membrane Lung for Extracorporeal Carbon Dioxide Removal ($ECCO_2R$): In Vitro Study

Luigi Vivona [1,†], Michele Battistin [2,†], Eleonora Carlesso [1,†], Thomas Langer [3,4,*], Carlo Valsecchi [5], Sebastiano Maria Colombo [1,5], Serena Todaro [1], Stefano Gatti [2], Gaetano Florio [1], Antonio Pesenti [1,5], Giacomo Grasselli [1,5] and Alberto Zanella [1,5]

1 Anesthesia and Critical Care, Department of Pathophysiology and Transplantation, University of Milan, 20122 Milan, Italy; luigi.vivona@unimi.it (L.V.); eleonora.carlesso@unimi.it (E.C.); sebastiano.colombo@gmail.com (S.M.C.); serena.todaro@outlook.it (S.T.); gaetano.florio@unimi.it (G.F.); antonio.pesenti@unimi.it (A.P.); giacomo.grasselli@unimi.it (G.G.); alberto.zanella1@unimi.it (A.Z.)
2 Center for Preclinical Research, Fondazione IRCCS Ca' Granda-Ospedale Maggiore Policlinico, 20122 Milan, Italy; battistin.michele@gmail.com (M.B.); stefano.gatti@policlinico.mi.it (S.G.)
3 Department of Anesthesia and Intensive Care Medicine, Niguarda Ca' Granda, 20162 Milan, Italy
4 Department of Medicine and Surgery, University of Milan-Bicocca, 20900 Monza, Italy
5 Dipartimento di Anestesia, Rianimazione ed Emergenza Urgenza, Fondazione IRCCS Ca' Granda-Ospedale Maggiore Policlinico, 20122 Milan, Italy; carlovalsecchi5@gmail.com
* Correspondence: thomas.langer@unimib.it; Tel.: +39-02-64448580; Fax: +39-02-55033230
† These authors equally contributed to the study.

Citation: Vivona, L.; Battistin, M.; Carlesso, E.; Langer, T.; Valsecchi, C.; Colombo, S.M.; Todaro, S.; Gatti, S.; Florio, G.; Pesenti, A.; et al. Alkaline Liquid Ventilation of the Membrane Lung for Extracorporeal Carbon Dioxide Removal ($ECCO_2R$): In Vitro Study. *Membranes* **2021**, *11*, 464. https://doi.org/10.3390/membranes11070464

Academic Editor(s): Gennaro Martucci; Antonio Arcadipane; Marco Giani

Received: 28 May 2021
Accepted: 20 June 2021
Published: 22 June 2021

Publisher's Note: MDPI stays neutral with regard to jurisdictional claims in published maps and institutional affiliations.

Copyright: © 2021 by the author. Licensee MDPI, Basel, Switzerland. This article is an open access article distributed under the terms and conditions of the Creative Commons Attribution (CC BY) license (http://creativecommons.org/licenses/by/4.0/).

Additional Methods:

Reaction between NaOH and CO_2

The mechanism of neutralization between NaOH and CO_2 depends on the concentration of the NaOH solution and on the pH value.

When NaOH is very diluted (pH<10) hydration of CO_2 occurs and H_2CO_3 is formed. Carbonic acid then reacts with NaOH to form sodium bicarbonate ($NaHCO_3$):

$CO_2 + H_2O = H_2CO_3$

$NaOH + H_2CO_3 = NaHCO_3 + H_2O$

When NaOH solution is highly concentrated one (pH>10), carbon dioxide directly reacts with NaOH forming bicarbonate. Bicarbonate further reacts with the alkali to form sodium carbonate (Na_2CO_3):

$NaOH + CO_2 = NaHCO_3$

$NaHCO_3 + NaOH = Na_2CO_3 + H_2O$

Thus, highly concentrated NaOH solutions may absorb a conspicuous amount of CO_2 while keeping PCO_2 almost down to zero.

When the concentration of the alkali solution is quite low compared to the amount of CO_2 added, the reaction proceeds via the formation of carbonic acid and PCO_2 increase. But the acidic oxide is not completely neutralized in this case.

Mathematical Model:

To simulate the effects of CO_2 absorption by aqueous NaOH we computed the solutions of a system of equations starting from standard mass-action, mass-conservation and electroneutrality laws.

We simulated a *closed* system with aqueous NaOH at varying concentrations and flows in which we introduced a CO_2. The species involved in the equilibrium were water, NaOH and CO_2.

Gaseous carbon dioxide dissolves in water (**CO_2dissolved**) and it forms carbonic acid (**H_2CO_3**):

$CO_2 + H_2O = H_2CO_3$

which dissociates in proton (**H^+**) and bicarbonate (**HCO_3^-**):

$H_2CO_3 = HCO_3^- + H^+$

The bicarbonate may furtherly dissociate in H^+ and carbonate ion (**CO_3^{2-}**):

$HCO_3^- = CO_3^{2-} + H^+$

The dissociated plus the undissociated forms of carbonic buffers are termed total CO_2 (**TCO_2**).

Accordingly:

$$\frac{[HCO_3^-]\times[H^+]}{[CO_2 dissolved]} = K_C \quad \textbf{(Equation S1)}$$

where K_c represents the carbonic dissociation constant, which, in plasma, is equal to ≈ $10^{-6.129}$=7.43E-7 (pKc ≈ 6.129),

$$\frac{[CO_3^{2-}]\times[H^+]}{[HCO_{3_2}^-]} = K_3 \quad \textbf{(Equation S2)}$$

Where K_3 is equal to ≈ $10^{-10.329}$ = 4.69E-11 (pK_3 ≈ 10.329)
and

$$[TCO_2] = [HCO_3^-] + [CO_3^{2-}] + [CO_2 dissolved] \quad \textbf{(Equation S3)}$$

Finally CO_2dissolved is equal to the product of solubility of CO_2 (α = 0.0307 mmol×L^{-1}×$mmHg^{-1}$) and partial pressure of CO_2:

$$[CO_2 dissolved] = \alpha \times PCO_2 \quad \textbf{(Equation S4)}$$

Water dissociation is characterized by the equation:

$$\frac{[H^+]\times[OH^-]}{[H_2O]} = K_W \quad \text{(}\textbf{Equation S5}\text{)}$$

where, at 37°C, K_W is equal to 4.3×10E-16, and [H_2O] is the molar concentration of water, i.e. 55.3 mol×L^{-1}. As K_W is relatively small the changes in water concentration are insignificant compared to the total water concentration. Therefore, it is commonly accepted that the product K_W × [H_2O] is constant (water ionic product, K'_W). This means that:

$$[H^+]\times[OH^-] = K'_W \approx 10E\text{-}14 \quad \text{(}\textbf{equation S6}\text{)}$$

Sodium hydroxide (NaOH) is a strong base almost completely dissociated in water into sodium ions (Na^+) and hydroxyl ions (OH^-). This means that the dissociation constant is much higher than 1 (K≈1.0000E+03, pK ≈ -3). Analogously to weak substances, we can refer to an hypothetical undissociated form as **NaOH** and we can define **Natot** as the sum of Na^+ and NaOH. Accordingly:

$$\frac{[Na^+]\times[OH^-]}{NaOH} = K_{NaOH} \quad \textbf{(Equation S7)}$$

where K_{NaOH} is the dissociation constant of strong electrolytes equilibrium;
and

$$[Na^+] = [Natot] - [NaOH] \quad \textbf{(Equation S8)}$$

Finally, according to electroneutrality law:

$$[Na^+] + [H^+] - [HCO_3^-] - 2\times[CO_3^{2-}] - [OH^-] \quad \text{(}\textbf{Equation S9}\text{).}$$

The final system was composed of equations S1, S2, S3, S4, S6, S7, S8 and S9 and was solved for [H^+], [OH^-], [HCO_3^-], [CO_3^{2-}], [CO_2dissolved], PCO_2, [Na^+], [NaOH]. Finally, pH was computed as:

$$pH=-log10[H^+] \quad \text{(}\textbf{Equation S10}\text{).}$$

We simulated a *closed* system with aqueous NaOH at varying concentrations (from 10 to 100 by 20 mmol × L^{-1}) in which we introduced CO_2 at different concentrations (from 0 to 100 by 5 mmol × L^{-1}).

The system was solved iteratively substituting incrementing of 0.001 pH values from 0 to the maximal pH (i.e. pH value of the solution at the current NaOH concentration when no CO_2 was added) and minimizing the difference between added and estimated TCO_2. Estimated TCO_2 was computed according to equation S3.

Computed variables

Bicarbonate ion concentration ($[HCO_3^-]$) was calculated from pH and PCO_2 according to the Henderson-Hasselbalch equation ($pH = pK + \log_{10} \frac{[HCO_3^-]}{\alpha \times PCO_2}$) where α=0.0307 mmol × L^{-1} × $mmHg^{-1}$ (solubility of CO_2 in plasma) [1,2] and pK = 6.129 (negative logarithm of the equilibrium constant) [2–4].

$$HCO_3^- = \alpha \times PCO_2 \times 10^{pH-pK}$$

Plasma carbon dioxide content (expressed in mmol × L^{-1}) from each side of the membrane was calculated according to the logarithmic form of the Henderson-Hasselbalch equation for CO_2 using the method published by Douglas et al. [5]:

$$TCO_2 = \alpha \times PCO_2 \times (1 + 10^{pH-pK})$$

Carbon dioxide transfer across the membrane, VCO_2 (expressed in mL× min^{-1}), was calculated from the transmembrane TCO_2 difference:

$$VCO_2 = (TCO_{2PRE} - TCO_{2POST}) \times blood\ flow\ \times 25.45$$

TCO_{2PRE} represents CO_2 content before the membrane while TCO_{2POST} is the CO_2 content after the membrane, blood flow is measured in L × min^{-1} and the conversion factor is in mL × $mmol^{-1}$.

membranes

MDPI

Additional Table

Table 1s

	NaOH	10	30	60	90	100	*P* Conc.	*P* PRE/ POST	*P* Int.
pH$^{\$}$	PRE	7.328 (7.323 — 7.333)	7.333 (7.324 — 7.336)	7.315 (7.300 — 7.322)	7.331 (7.314 — 7.346)	7.331 (7.327 — 7.334)	0.044	<0.001	0.005
	POST	7.577 (7.485 — 7.643)*‖#	7.732 (7.651 — 7.753)*#	7.862 (7.790 — 7.893)*	7.906 (7.870 — 7.948)*	7.913 (7.885 — 7.943)*			
	Difference	0.249 (0.152 — 0.321)°§‖#	0.396 (0.325 — 0.418)§‖#	0.557 (0.487 — 0.574)	0.575 (0.556 — 0.602)	0.585 (0.556 — 0.612)	<0.001		
PCO_2 (mmHg)$^{\$}$	PRE	59.4 (58.4 — 60.4)	58.4 (57.6 — 58.8)	59.1 (58.0 — 61.4)	59.0 (57.1 — 60.9)	59.5 (58.6 — 60.7)	0.100	<0.001	0.042
	POST	27.3 (21.9 — 35.4)*‖#	16.2 (15.1 — 21.4)*	12.0 (11.0 — 15.0)*	11.4 (10.2 — 12.4)*	12.4 (11.3 — 13.1)*			
	Difference	-32.2 (-38.6 — -23.1)§‖#	-41.4 (-43.1 — -36.8)	-47.7 (-49.5 — -44)	-47.8 (-48.6 — -47)	-48.2 (-48.4 — -46.6)	<0.001		
PO_2 (mmHg)	PRE	157.0 (153.5 — 159.5)§#	153.5 (151.0 — 157.5)#	145.5 (145.0 — 150.5)#	147.5 (146.0 — 151.0)#	125.0 (124.5 — 125.0)	<0.001	0.676	0.006
	POST	159.0 (149.5 — 176.0)§‖#	151.0 (142.0 — 170.0)§‖#	136.0 (129.5 — 152.5)#	140.0 (128.0 — 156.0)#	109.2 (97.2 — 126.5)*			
	Difference	2.0 (-4.0 — 16.5)#	-5.0 (-13.0 — 16.5)	-9.5 (-16.0 — 2.5)	-7.5 (-18.0 — 5.0)	-15.3 (-27.3 — 1.5)	0.006		
K^+ ($mEq \times L^{-1}$)	PRE	4.3 (4.3 — 4.3)§#	4.4 (4.4 — 4.5)	4.5 (4.5 — 4.5)	4.4 (4.4 — 4.5)	4.5 (4.4 — 4.5)	0.005	0.005	0.366
	POST	4.3 (4.2 — 4.3)*§#	4.3 (4.3 — 4.3)*	4.4 (4.4 — 4.5)*	4.3 (4.3 — 4.4)*	4.4 (4.3 — 4.4)*			
	Difference	0.0 (-0.1 — 0.0)	-0.1 (-0.2 — -0.1)	0.0 (-0.1 — 0.0)	-0.1 (-0.1 — -0.1)	-0.1 (-0.1 — -0.1)	0.366		
Na^+ ($mEq \times L^{-1}$)	PRE	140.5 (140.0 — 141.0)	141.5 (140.5 — 143.5)	140.5 (139.5 — 141.5)	140.0 (139.0 — 142.5)	142.5 (142.0 — 143.0)	0.429	<0.001	0.263
	POST	138.5 (137.5 — 139.0)*	137.5 (137.0 — 138.5)*	137.0 (136.5 — 139.0)*	137.0 (136.0 — 139.0)*	139.5 (139.0 — 140.0)*			

<table>
<tr><td></td><td>Difference</td><td>-2.0 (-3.0 — -1.5)</td><td>-4.0 (-5.5 — -3.0)</td><td>-2.5 (-4.0 — -1.5)</td><td>-3.0 (-3.5 — -3.0)</td><td>-3.0 (-3.0 — -3.0)</td><td>0.263</td><td></td><td></td></tr>
<tr><td rowspan="3">Ca^{++} (mEq×L^{-1})$</td><td>PRE</td><td>1.1 (1.1 — 1.1)°§||#</td><td>1.2 (1.2 — 1.2)§||#</td><td>1.3 (1.3 — 1.4)</td><td>1.3 (1.3 — 1.4)</td><td>1.4 (1.4 — 1.4)</td><td><0.001</td><td><0.001</td><td><0.001</td></tr>
<tr><td>POST</td><td>1.0 (1.0 — 1.0)*§||#</td><td>1.1 (1.0 — 1.1)*§||#</td><td>1.2 (1.2 — 1.2)*#</td><td>1.2 (1.1 — 1.2)*#</td><td>1.3 (1.3 — 1.3)*</td><td></td><td></td><td></td></tr>
<tr><td>Difference</td><td>-0.1 (-0.1 — -0.1)°§||</td><td>-0.1 (-0.1 — -0.1)||</td><td>-0.1 (-0.2 — -0.1)#</td><td>-0.2 (-0.2 — -0.2)#</td><td>-0.1 (-0.1 — -0.1)</td><td><0.001</td><td></td><td></td></tr>
<tr><td rowspan="3">Cl^- (mEq×L^{-1})$</td><td>PRE</td><td>107.0 (106.5 — 107.0)#</td><td>106.0 (104.5 — 106.0)#</td><td>108.0 (106.5 — 108.0)</td><td>108.0 (105.5 — 108.5)</td><td>110.0 (110.0 — 110.0)</td><td>0.017</td><td><0.001</td><td>0.190</td></tr>
<tr><td>POST</td><td>110.0 (108.5 — 111.5)*#</td><td>110.5 (109.5 — 111.0)*#</td><td>112.5 (110.5 — 113.0)*</td><td>112.0 (109.5 — 113.0)*</td><td>114.0 (114.0 — 114.0)*</td><td></td><td></td><td></td></tr>
<tr><td>Difference</td><td>3.0 (2.0 — 4.5)</td><td>5.0 (4.5 — 5.5)</td><td>4.5 (4.0 — 5.0)</td><td>4.0 (4.0 — 4.5)</td><td>4.0 (4.0 — 4.0)</td><td>0.135</td><td></td><td></td></tr>
<tr><td rowspan="3">Lac (mEq×L^{-1})$</td><td>PRE</td><td>2.3 (2.3 — 2.4)°§||#</td><td>2.5 (2.5 — 2.6)§#</td><td>3.1 (3.1 — 3.2)||</td><td>2.7 (2.6 — 2.8)#</td><td>4.4 (4.2 — 4.5)</td><td><0.001</td><td>0.517</td><td>0.736</td></tr>
<tr><td>POST</td><td>2.4 (2.3 — 2.4)°§||#</td><td>2.7 (2.4 — 2.9)§#</td><td>3.1 (3.1 — 3.2)||</td><td>2.6 (2.6 — 2.7)#</td><td>4.3 (4.2 — 4.5)</td><td></td><td></td><td></td></tr>
<tr><td>Difference</td><td>0.0 (0.0 — 0.1)</td><td>0.1 (-0.1 — 0.3)</td><td>0.0 (-0.1 — 0.1)</td><td>0.0 (-0.1 — 0.0)</td><td>0.0 (-0.1 — 0.0)</td><td>0.842</td><td></td><td></td></tr>
<tr><td rowspan="3">Hb (g×dL^{-1})</td><td>PRE</td><td>13.90 (13.90 — 13.95)§||#</td><td>14.10 (14.00 — 14.20)§||#</td><td>10.40 (10.35 — 10.45)#</td><td>10.55 (10.50 — 10.60)#</td><td>9.20 (9.10 — 9.30)</td><td><0.001</td><td>0.651</td><td>0.183</td></tr>
<tr><td>POST</td><td>13.85 (13.80 — 13.95)§||#</td><td>14.05 (13.85 — 14.20)§||#</td><td>10.50 (10.50 — 10.50)#</td><td>10.55 (10.50 — 10.80)#</td><td>9.25 (9.15 — 9.35)</td><td></td><td></td><td></td></tr>
<tr><td>Difference</td><td>-0.10 (-0.10 — 0.00)</td><td>0.00 (-0.20 — 0.05)</td><td>0.10 (0.05 — 0.15)</td><td>0.00 (0.00 — 0.20)</td><td>0.05 (0.00 — 0.10)</td><td>0.183</td><td></td><td></td></tr>
<tr><td rowspan="3">HCO_3^- (mmol×L^{-1})</td><td>PRE</td><td>28.8 (28.7 — 29)</td><td>28.5 (28.1 — 28.7)</td><td>27.9 (27.7 — 27.9)</td><td>28.8 (28.6 — 28.9)</td><td>29.1 (28.7 — 29.5)</td><td><0.001</td><td><0.001</td><td>0.021</td></tr>
<tr><td>POST</td><td>23.2 (21.9 — 24.5)*°§||</td><td>19.9 (19.5 — 21.3)*#</td><td>20 (19.6 — 20.8)*#</td><td>20.7 (20.4 — 21)*#</td><td>22.7 (22.2 — 23.1)*</td><td></td><td></td><td></td></tr>
<tr><td>Difference</td><td>-5.6 (-7.1 — -4.2)</td><td>-8.6 (-9.2 — -6.8)</td><td>-7.9 (-8.3 — -6.9)</td><td>-7.9 (-8.4 — -7.7)</td><td>-6.4 (-6.6 — -6.3)</td><td>0.021</td><td></td><td></td></tr>
<tr><td rowspan="3">plasma TCO_2 (mmol×L^{-1})</td><td>PRE</td><td>30.7 (30.5 — 30.8)</td><td>30.2 (29.9 — 30.5)</td><td>29.7 (29.5 — 29.8)</td><td>30.6 (30.5 — 30.6)</td><td>30.9 (30.5 — 31.3)</td><td><0.001</td><td><0.001</td><td>0.019</td></tr>
<tr><td>POST</td><td>24 (22.6 — 25.6)*°§||</td><td>20.4 (19.9 — 21.9)*#</td><td>20.3 (20 — 21.3)*#</td><td>21.1 (20.8 — 21.3)*#</td><td>23.1 (22.5 — 23.5)*</td><td></td><td></td><td></td></tr>
<tr><td>Difference</td><td>-6.6 (-8.2 — -4.9)||</td><td>-9.9 (-10.5 — -7.9)</td><td>-9.4 (-9.8 — -8.3)</td><td>-9.4 (-9.8 — -9.2)</td><td>-7.8 (-8.1 — -7.7)</td><td>0.019</td><td></td><td></td></tr>
</table>

<table>
<tr><td>VCO_2 (mL×min^{-1})</td><td></td><td>73.9 (54.3 — 91.8)||</td><td>109.7 (88.3 — 117.1)</td><td>104.5 (92.8 — 108.7)</td><td>104.3 (102.4 — 109.0)</td><td>87.2 (85.8 — 89.9)</td><td>0.019</td><td></td><td></td></tr>
</table>

Table 1s: Safety and feasibility tests results. Abbreviations: PCO_2, partial pressure of carbon dioxide; PO_2, partial pressure of oxygen; Na^+, sodium; K^+, potassium; Ca^{++}, calcium; Cl^-, chloride; Lac, Lactate; Hb, Hemoglobin; $HCO3^-$, bicarbonate, TCO_2, total CO_2 content, $\dot{V}CO_2$, amount of carbon dioxide removed by the membrane lung. Data are expressed median (IQR); Differences were computed as POST values – PRE values. P: P values of two-way ANOVA RM or two-way ANOVA RM on ranks ($^\$$) for PRE and POST values (P PRE/POST) and NaOH concentration (P Conc.) and interaction (P int.); one-way ANOVA RM or one-way ANOVA RM on ranks were applied for difference values. Post-hoc analysis with Bonferroni or Tukey corrections: * P<0.05 vs pre; ° P<0.05 vs 30; § P<0.05 vs 60; || P<0.05 vs 90; # P<0.05 vs 100.

References

1. Austin, W.H.; Lacombe, E.; Rand, P.W.; Chatterjee, M. Solubility of Carbon Dioxide in Serum from 15 to 38 C. *J. Appl. Physiol.* **1963**, *18*, 301–304, doi:10.1152/jappl.1963.18.2.301.
2. Constable, P.D. Total Weak Acid Concentration and Effective Dissociation Constant of Nonvolatile Buffers in Human Plasma. *J. Appl. Physiol.* **2001**, *91*, 1364–1371, doi:10.1152/jappl.2001.91.3.1364.
3. Harned, H.S.; Bonner, F.T. The First Ionization of Carbonic Acid in Aqueous Solutions of Sodium Chloride. *J. Am. Chem. Soc.* **1945**, *67*, 1026–1031, doi:10.1021/ja01222a037.
4. Putnam, R.W.; Roos, A. Which Value for the First Dissociation Constant of Carbonic Acid Should Be Used in Biological Work? *Am. J. Physiol.-Cell Physiol.* **1991**, *260*, C1113–C1116, doi:10.1152/ajpcell.1991.260.5.C1113.
5. Douglas, C.G.; Haldane, J.S. (From the Physiological Laboratory, Oxford, and the Institute of General Pathology, Copenhagen.). 28.
